# Evaluating the 2024 dog oral rabies vaccination campaign in the Zambezi region, Namibia using GIS and household surveys

**DOI:** 10.1038/s41598-026-38405-x

**Published:** 2026-03-16

**Authors:** Conrad M. Freuling, Mainelo Beatrice Shikongo, Frank Busch, Sarah Gottlieb, Reinhold Haimbodi, Naindji Haindongo, Chantal Hansen, Juliet Kabajani, Joseph Kapapero, Muesee Kasaona, Mattia Marconcini, Jeremia Namusheshe, Nzwana Silume, Tenzin Tenzin, Ad Vos, Thomas Müller

**Affiliations:** 1 Institute of Molecular Virology and Cell Biology, Friedrich-Loeffler-Institut (FLI), WOAH Reference Laboratory for Rabies, Greifswald-Insel Riems, Germany; 2 State Veterinary Office, Ministry of Agriculture, Water and Land Reform, Directorate of Veterinary Services, Zambezi Region, Katima Mulilo, Namibia; 3 Institute of International Animal Health/One Health, Friedrich-Loeffler-Institut (FLI), Greifswald-Insel Riems, Germany; 4 State Veterinary Office, Ministry of Agriculture, Water and Land Reform, Directorate of Veterinary Services, Kavango East Region, Nkurunkuru, Namibia; 5 Ministry of Agriculture, Water and Land Reform, Directorate of Veterinary Services, Windhoek, Namibia; 6 Central Veterinary Laboratory (CVL), Ministry of Agriculture, Water and Land Reform, Directorate of Veterinary Services, Windhoek, Namibia; 7 German Aerospace Center–DLR, Cologne, Germany; 8 World Organisation for Animal Health (WOAH), Sub-Regional Representation for Southern Africa, Gaborone, Botswana; 9 CEVA Sante Animale, Libourne, France

**Keywords:** Africa, Dogs, Namibia, Oral vaccination, Rabies, SPBN GASGAS, GIS, Post vaccination survey, Bite incidence, Diseases, Health care, Microbiology

## Abstract

The 2024 oral rabies vaccination (ORV) campaign in Namibia’s Zambezi region aimed to address persistent rabies transmission through a large-scale effort combining direct dog vaccination and owner-assisted distribution. A total of 9,393 baits were distributed, and the campaign demonstrated feasibility and operational efficiency, achieving an estimated vaccination coverage of 47.9% of dogs in just four days, increasing to 56.8% when accounting for prior parenteral vaccination efforts. Spatial analysis revealed an average vaccination coverage of 60% across 10 × 10 km grid cells, and encompassed 81% of the human population of the Zambezi region within the vaccination coverage area. Post-vaccination surveys indicated a rise in vaccination rates (54.5% of dogs vaccinated, up from 18.9%) and lower presence of rabies. Improved education and awareness campaigns, targeted interventions in low-coverage areas, and enhanced surveillance are recommended to further reduce rabies incidence. The findings highlight the promise and proficiency of ORV campaigns in resource-constrained settings and demonstrate that its application can be key for sustained rabies control efforts in Namibia.

**Supplementary Information:**

The online version contains supplementary material available at 10.1038/s41598-026-38405-x.

## Introduction

**Fig. 1:**
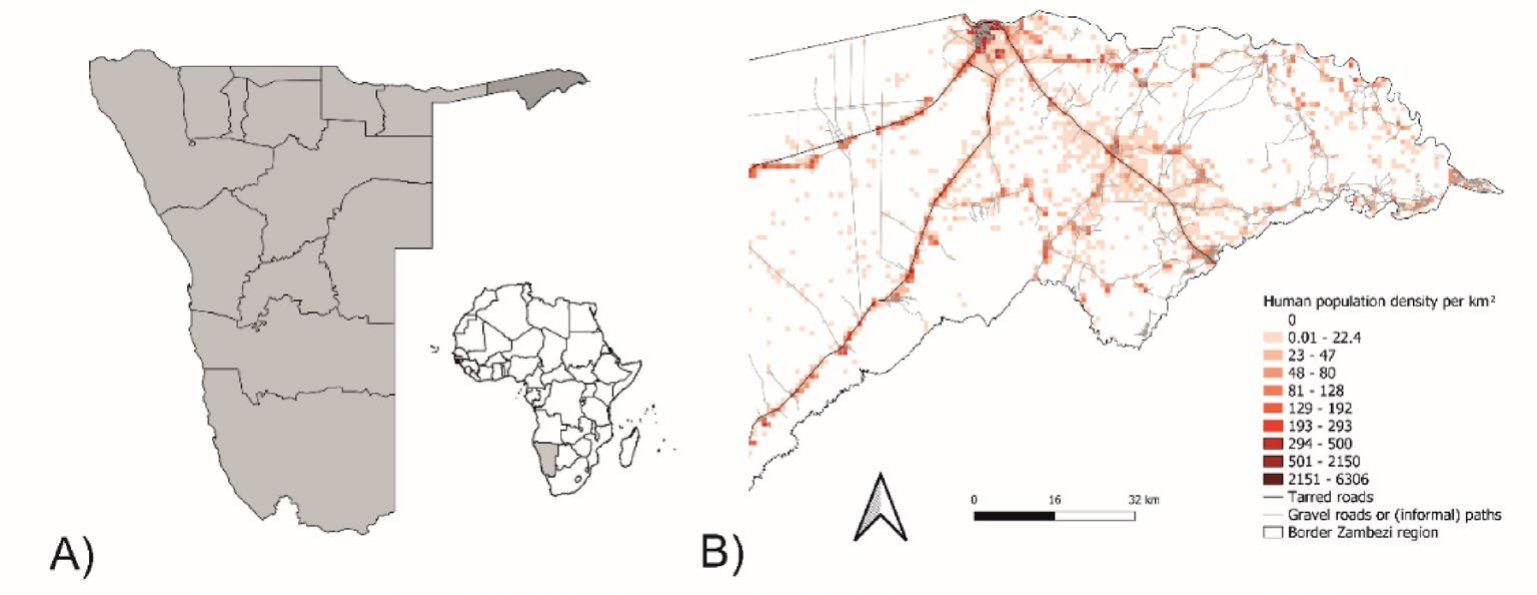
(**A**) Map of Namibia with the administrative boundaries of regions and the Zambezi region highlighted. (**B**) Details of the Zambezi region indicating the human population density per km² and road networks. The population density was calculated from the High Resolution Settlement Layer (HRSL) from Meta (formerly facebook). The maps were created using QGIS (version 3.40.2; QGIS Development Team, 2024; https://qgis.org/).

Rabies remains a significant public health challenge in many parts of the world^1^, particularly in regions where interactions between humans and domestic dogs are frequent, and access to veterinary and public health resources is limited. In Namibia, dog-mediated rabies is endemic in the Northern Communal Areas (NCAs) causing human cases^2^. NCAs of Namibia refer to the region north of the veterinary cordon fence (also known as the “Red Line”), encompassing the country’s predominantly communal farming regions. These areas include the Omusati, Oshana, Ohangwena, Oshikoto, Kavango East, Kavango West, and Zambezi regions (Fig. 1). Namibia has implemented a national dog rabies control strategy which was one of the first national rabies plans to be endorsed by the World Organisation for Animal Health (WOAH) in 2021, validating its quality as well as Namibia’s commitment to regular reporting on rabies control progress. One key element is mass dog vaccinations being applied in recent years^3,4^. Additionally, oral rabies vaccination (ORV) has emerged as a promising tool to supplement traditional parenteral vaccination efforts^5,6^, offering a feasible approach to reaching free-roaming and hard-to-handle dog populations. In Namibia, studies on immunogenicity^7^ as well as field trials^8^ showed the functionality, acceptance and feasibility of oral rabies vaccine baits for vaccinating dogs. These experiences partly built the foundations for internationally (WOAH, FAO and WHO) accepted guidelines on ORV use^9^.

The Zambezi region is one area in Namibia’s NCAs where both environmental and logistical factors, such as the presence of other diseases (Foot and mouth disease, Contagious bovine pleuropneumonia, etc.), and the presence of flood areas that require four-wheel-drive pickup trucks or boats, pose challenges to achieving effective rabies control (Fig. 1). In a field study in the Zambezi region in 2022, an ORV-only approach was successfully used as an emergency response model to rabies incidences in dogs which proved to be highly effective^10^.

Building upon these previous experiences and leveraging advancements in field logistics, another ORV campaign was conducted in 2024, aiming to assess the feasibility of large-scale oral bait distribution, evaluate vaccination coverage, and measure public acceptance and awareness of ORV as a strategy. The integration of geospatial tools and mobile data collection platforms provided a robust framework for monitoring and analyzing campaign outcomes, ensuring that the efforts were data-driven and targeted. In addition to detailing the operational aspects of the campaign, this study also examined the impact of vaccination efforts on the occurrence of rabies and public attitudes toward rabies control measures in comparison with the baseline data from a 2021 Knowledge, Attitudes, and Practices (KAP) study^11^. A cross-sectional post-vaccination survey provided critical insights into the challenges and barriers to achieving higher vaccination coverage and the broader implications for rabies control and prevention in the region.

## Materials and methods

### Vaccination campaign

This vaccination campaign was planned and carried out largely as previously described^8^. In brief, the ORV field trial took place in the Zambezi region covering 14,745 km² of Namibia, in areas selected after consultation with the Directorate of Veterinary Services (DVS), based on human population, available infrastructure and logistical considerations, in June 2024 (Fig. 1B, Supplementary Fig. 1).

The vaccination of dogs was conducted using Rabitec^®^ (Ceva Sante Animale, Libourne, France). This third-generation oral rabies vaccine is based on the SPBN GASGAS virus strain, which is licensed for use in foxes, raccoon dogs, and dogs in Europe^12^. A total of 10,000 vaccine baits were purchased and shipped directly from the manufacturer to the importing company, Swavet in Windhoek, Namibia, in compliance with national import regulations and IATA guidelines.

Ten vaccination teams, each consisting of a driver/data collector and a vaccinator, used four-wheel-drive pickup trucks equipped with cooler bags, gloves, trash bags, hand sanitizers, and informational leaflets. Prior to the field trial, a two-hour training workshop was conducted, during which staff were briefed on the objectives of the trial, proper handling of the vaccine baits, safety protocols, techniques for approaching free-roaming dogs, best practices for offering vaccine baits, and data collection procedures. Dog owners were informed about the ORV initiative through a leaflet provided in both English (the official language) and Lozi (the local language), which also included an emergency contact number for reporting any adverse events. The ORV campaigns in individual regions were announced on local radio the day before.

In cases where dogs could not be vaccinated directly, such as when dogs were wary or not within their usual home-range, baits were provided to the owner, with strict instructions on how and when to administer the baits, following the guidelines within the distributed leaflets. Initially, four working days were planned for bait distribution, with an additional four days allocated for further distribution of remaining baits by DVS staff outside of the planned schedule. To collect vaccination data and manage the project—such as navigating within designated boundaries, sharing real-time team locations, and assessing survey progress—a smartphone application was used, which integrates with the WVS web-based backend platform, as previously described^13,14^ .

### Post vaccination monitoring

A cross-sectional study was conducted in December 2024 in Zambezi and included the entire implementation area. Door-to-door surveys were conducted by a total of 10 enumerators in teams of two over a period of 5 days. The survey was designed and conducted based on a previous KAP study^11^. The survey aimed at assessing public awareness, vaccination practices, rabies presence, bite incidence, attitudes towards ORV as a tool and the reported vaccine uptake, also in relation to the previous assessment in 2022^8^. While for the majority of questions, closed multiple-choice questions using radio buttons were used, for the ORV attitude a 5-point Likert scale – from strong agreement to strong disagreement, was implemented. The questionnaire is available in the Supplementary Materials (S1 Table).

The target number of surveyed households was 385 in villages/settlements around so-called “crush pens”, animal holding facilities which are also used as a veterinary epidemiological unit. To achieve the sample size, survey teams were required to interview at least 15 households in the vicinity of selected crush pens. In order to take potentially incomplete or compromised data sets into account, the sample size was increased to 20 households per crush pen. Verbal informed consent was obtained before entering the data into the app, and no personal data such as names of the participants were collected.

### Data analysis, GIS and statistical evaluation

Data collected during both the vaccination study (S2 Table) and the survey (S3 Table) were downloaded from WVS software in comma-separated value files. Additionally, data from a parenteral vaccination campaign in 2023 captured using the GARC mobile app^4^ were also downloaded and used.

Human population and household data from the 2016 Inter Censal Demographic Survey (NIIDS)^15^ were used as a baseline to estimate the total dog population in the NCA by region and constituency according to recent literature^16^. The dog population was then projected for 2021 using the annual growth rate for the human population according to the Namibian Statistics Agency. The human population density at the location of the individual survey was derived from the High Resolution Population Density Geotiff Maps (Data for Good at Meta, https://dataforgood.facebook.com/dfg/tools/high-resolution-population-density-maps). Additionally, building footprints from Google (https://sites.research.google/gr/open-buildings/) were used and associated with open street map (OSM) use (residential/non-residential) and joined with the 2021 population data. In order to derive the spatial coverage of our campaign, the locations where dogs were offered a bait were buffered by 500 m assuming that people would either bring their dogs for vaccination or dogs would have been sighted and vaccinated. All houses and the respective population data within this buffer was then calculated, regarded as being covered and contrasted to the overall population. All GIS analyses were performed in QGIS-Version 3.22.0-Białowieża (https://qgis.org/).

Confidence intervals (95%) for the survey derived vaccination coverage were calculated using the Wilson-Brown method. One-way ANOVA followed by Dunnett’s multiple comparison tests using a single pooled variance was used to identify statistically significant differences in vaccination efficiencies between studies. All calculations were performed in Prism 10.4.0 (GraphPad Software, Inc., https://www.graphpad.com).

### Ethical and legal considerations

The ORV field trial and the post vaccination monitoring survey was planned in close cooperation with the Namibian Directorate of Veterinary Services (DVS) within the Ministry of Agriculture, Water, Forestry and Land Reform (MAWLR). Import approval (Permit no: Path 08/2024, Ref.: V13/1/3/2/1/2) of the vaccine was conducted as described before^8^. The oral rabies field study in the Zambezi region and its ethical clearance were approved by the Chief Veterinary Officer of the Republic of Namibia in agreement with the medicines registrar at the Namibian Medicines Regulatory Council (NMRC, Clearance Number 28.08.2023/CC/V2023-0018/PS). All methods were carried out in accordance with relevant guidelines and regulations. As part of the official rabies vaccination campaign, animal ethics considerations did not apply.

## Results

### Vaccination campaign

**Fig. 2:**
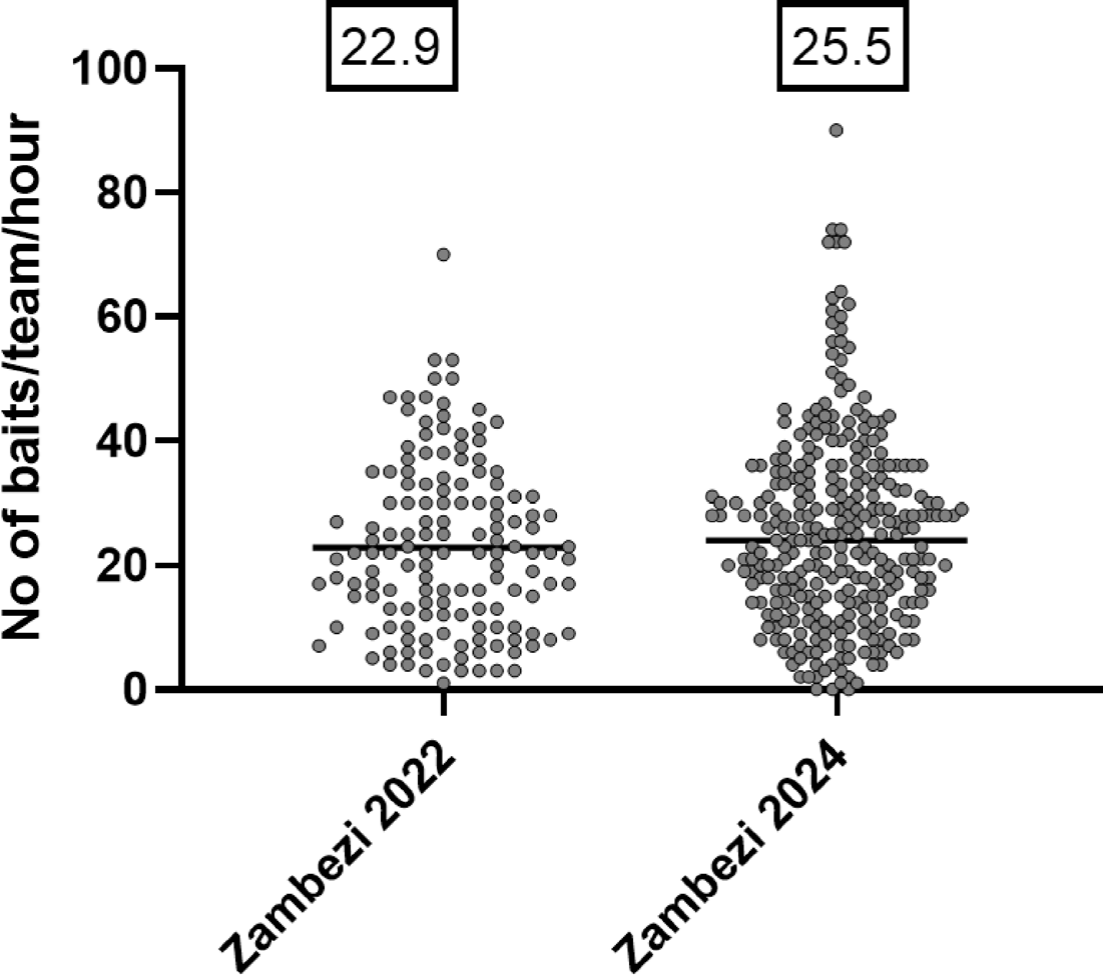
Scatter plots showing the number of baits distributed per team and hour, comparing the 2024 campaign to the previous emergency vaccination effort in 2022^10^. The mean (black horizontal lines) is indicated above the scatter plot.

During the four planned days of the ORV campaign, a total of 6,562 baits were directly administered to dogs, and an additional 770 baits were distributed indirectly through dog owners. This resulted in a team distribution of 164 baits per day without owner involvement, and 183 baits per day with owner distribution. On average, each team distributed 25.5 baits per hour (Fig. 2).

**Fig. 3:**
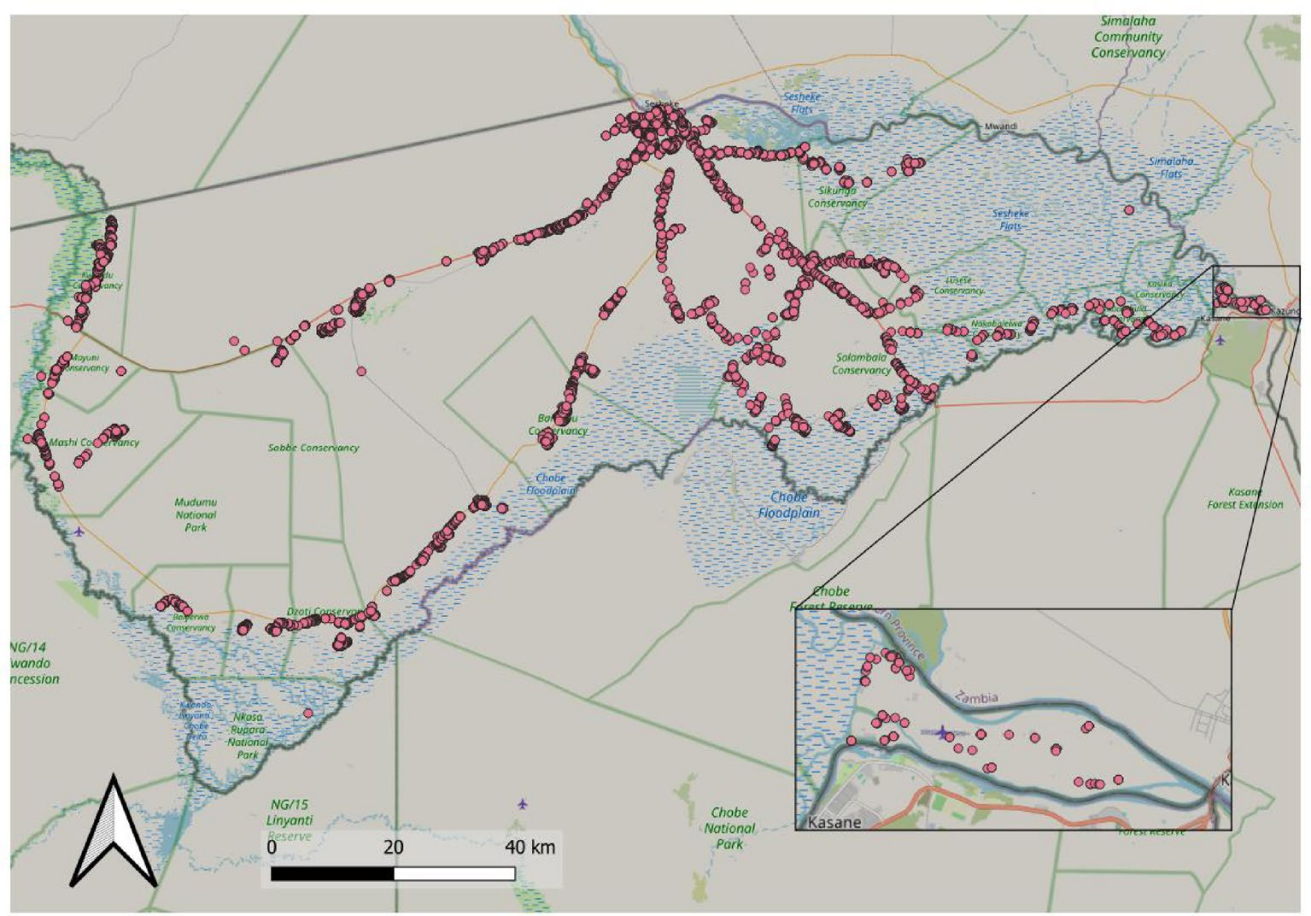
Map of the Zambezi region, with locations of orally vaccinated dogs (*N* = 9393) indicated. Impalila Island is shown as insert. The map was created using QGIS (version 3.40.2; QGIS Development Team, 2024; https://qgis.org/) with OpenStreetMap data as the basemap (© OpenStreetMap contributors; https://www.openstreetmap.org).

In addition, baiting was conducted on a separate day over the weekend to include Impalila Island, a small island in the Zambezi River. Furthermore, 1,775 remaining baits from the campaign were utilized by the Directorate of Veterinary Services (DVS) in areas not covered during the initial campaign. As these activities were partially linked to other DVS duties, the data were excluded from the effectiveness analysis. Overall, a total of 9,393 oral baits were offered in the Zambezi region in 2024 (Fig. 3), with 1,777 (19%) of these baits being handed to owners for distribution.

**Fig. 4:**
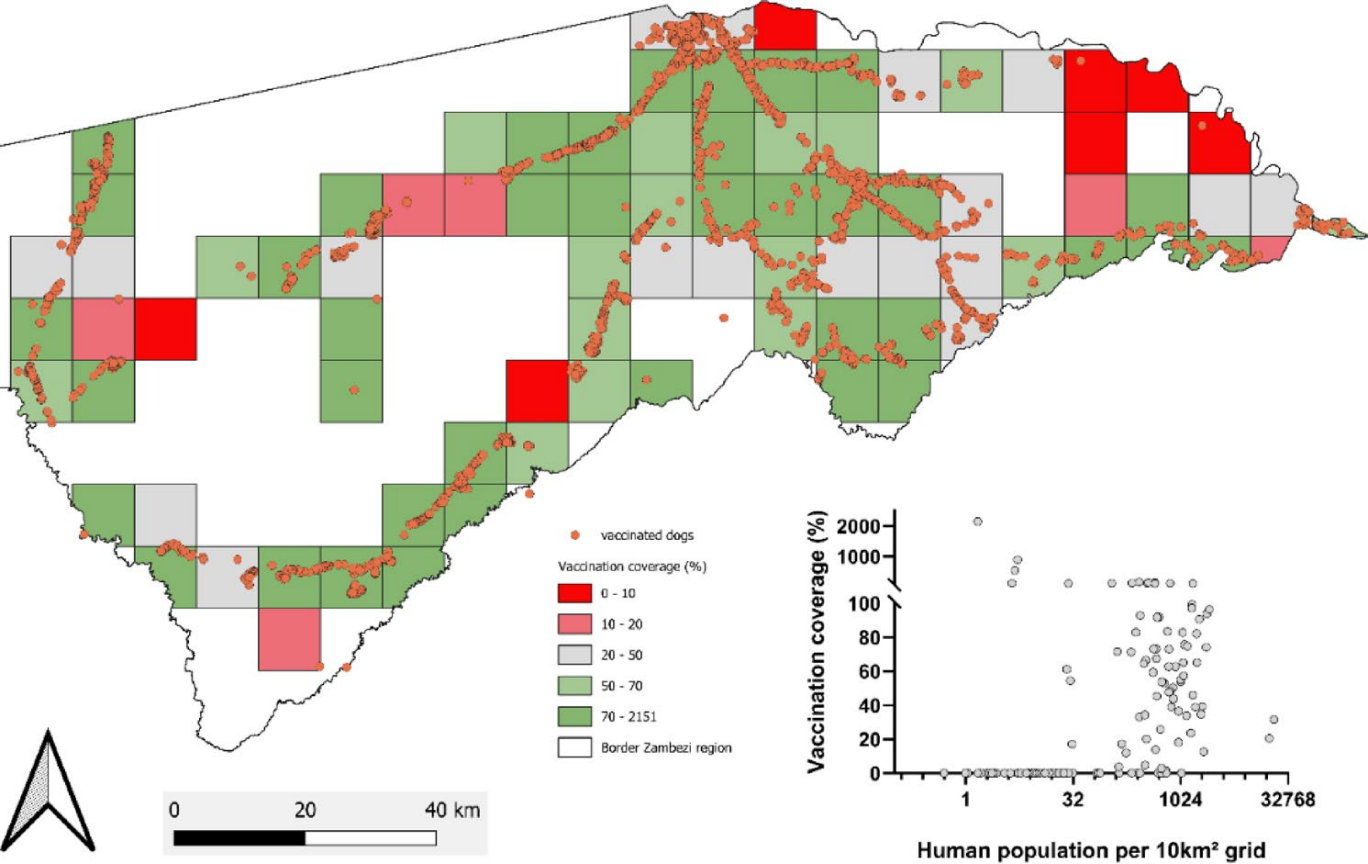
Zambezi region, overlaid with a 10 × 10 km grid and vaccination point data from the oral dog vaccination campaign. Human population data per grid was derived from the High Resolution Population Density Maps (META), and the dog population was estimated using the human-to-dog ratio of 5.4^11^. Vaccination coverage (%) per grid cell was calculated as the number of dogs vaccinated divided by the estimated dog population, and displayed for grid cells > 500 people with a color code. Insert: Scatter plot of individual vaccination coverages per grid cell in relation to the respective human population density per grid cell. Values above 100% reflect artefacts due to spatial mismatches between vaccination counts and population estimates: in some grids, vaccination numbers were higher than the dog population inferred from human population estimates, yielding coverage values > 100%. The map was created using QGIS (version 3.40.2; QGIS Development Team, 2024; https://qgis.org/).

Several methods were employed to estimate vaccination coverage following the ORV campaign. Using the human: dog ratio (HDR) of 5.4 from a prior KAP study in 2021^11^, the estimated dog population in the Zambezi region was 19,629, which resulted in an ORV vaccination coverage of 47.9%. Assuming that no dog has been vaccinated twice, the inclusion of 1,763 dogs vaccinated as part of the last mass dog vaccination campaign in 2024, an overall coverage of up to 56.8% can be recorded. When vaccination coverage of the ORV campaign was calculated for each 10 × 10 km grid cell, the mean coverage was 60% (95% CI: 28–92%). Only a few grid cells with populations greater than 500 showed vaccination coverages below 20% (Fig. 4).

Spatially, when vaccination points were buffered by a 500-meter radius and all homesteads within this buffer were considered as covered, the total number of buildings within the coverage area accounted for 73% of all buildings in the Zambezi region. Additionally, 81% of the total human population was covered (Supplementary Fig. 2).

### Post vaccination survey

As part of the post-vaccination survey, a total of 460 households were interviewed across most areas of the Zambezi region (Supplementary Fig. 3). The characteristics of the respondents were similar to those observed in the 2021 KAP study. The majority of surveys were conducted in rural areas, with farming being the most common occupation (Table 1).

Compared to the previous survey, there was a notable increase in the number of households with dogs: 70.2% reported having dogs in 2024, up from 47.1% in 2021. Vaccination rates also rose significantly, with 59.4% of households and 54.5% (95% CI:50.7–58.2) of dogs vaccinated in 2024, compared to 21.5% (95% CI: 16.6–27.4) and 18.9% (95% CI: 15.7–22.7) in 2021, respectively. Among the vaccinated dogs, 86.8% received the oral bait vaccine.

When asked about reasons for not vaccinating their dogs, 70% of respondents cited lack of awareness, while 18.4% mentioned the dog’s age (i.e. too young). Other reasons, such as absence or lack of time (6.4%), distance (4.3%), and handling difficulties (1.4%), were less frequently reported. In 2021, the most common reason was also lack of awareness (67.5%), followed by distance (14.1%), age (8.9%), handling issues (4.7%), and absence/no time (4.7%).

**Fig. 5:**
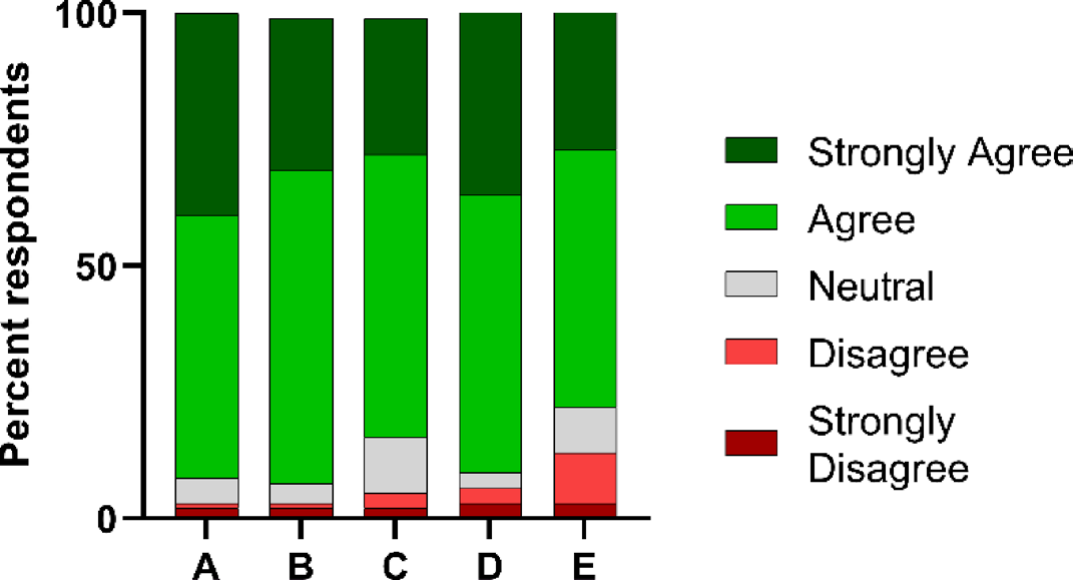
Cumulative assessment of the attitude towards oral rabies vaccination using the Likert scale, with every response being valued as one. The questions were: (**A**) Do you think vaccination with an oral bait is a good tool? (**B**) My dogs could only be vaccinated by oral baits (**C**) The best way is to combine parenteral vaccination of puppies with oral vaccination of free roaming dogs (**D**) Oral rabies vaccination should be included in the vaccination strategy, and (**E**) I would also pick up baits from a central source (store, headman, etc.) to vaccinate my own dogs.

An analysis of attitudes towards the oral rabies vaccination (ORV) showed a very positive perception (Fig. 5).

**Table 1:** Results from the 2021 and 2024 surveys in the Zambezi region.

	2021		2024	
Households	473		460	
**Variables**	**N**	**(%)**	**N**	**(%)**
Education				
Primary level	84	18.5	99	21.5
Secondary level	232	51	181	39.4
Graduate	58	12.7	44	9.6
Others	0	0	1	0.2
Not attended school	99	21.8	135	29.4
Occupation				
Farmer	195	42.9	207	45.0
Business	47	10.3	20	4.4
Government employee	48	10.5	40	8.7
Corporate employee	15	3.3	0	0.0
Student	47	10.3	33	7.2
Unemployed	116	25.5	143	31.1
Others	5	1.1	3	0.7
Residence				
Rural	413	87.3	398	86.5
Urban	60	12.7	62	13.5
Livestock ownership	319	67.4	355	77.2
Dog ownership	223	47.1	323	70.2
Dogs/HH	1.03		1.5	
Dogs/DOHH	2.2		2.1	
Dogs	486		670	
Humans	2,775		2,523	
HDR	5.7		3.8	
Dogs vaccinated	92	18.9 (95% CI: 15.7–22.7)	365	54.5 (95% CI:50.7–58.2)
HH vaccinated	48	21.5 (95% CI: 16.6–27.4)	192	59.4 (95% CI:54.0-64.7)

HH=household, DOHH = dog-owning household, HDR=human:dog ratio.

**Fig. 6:**
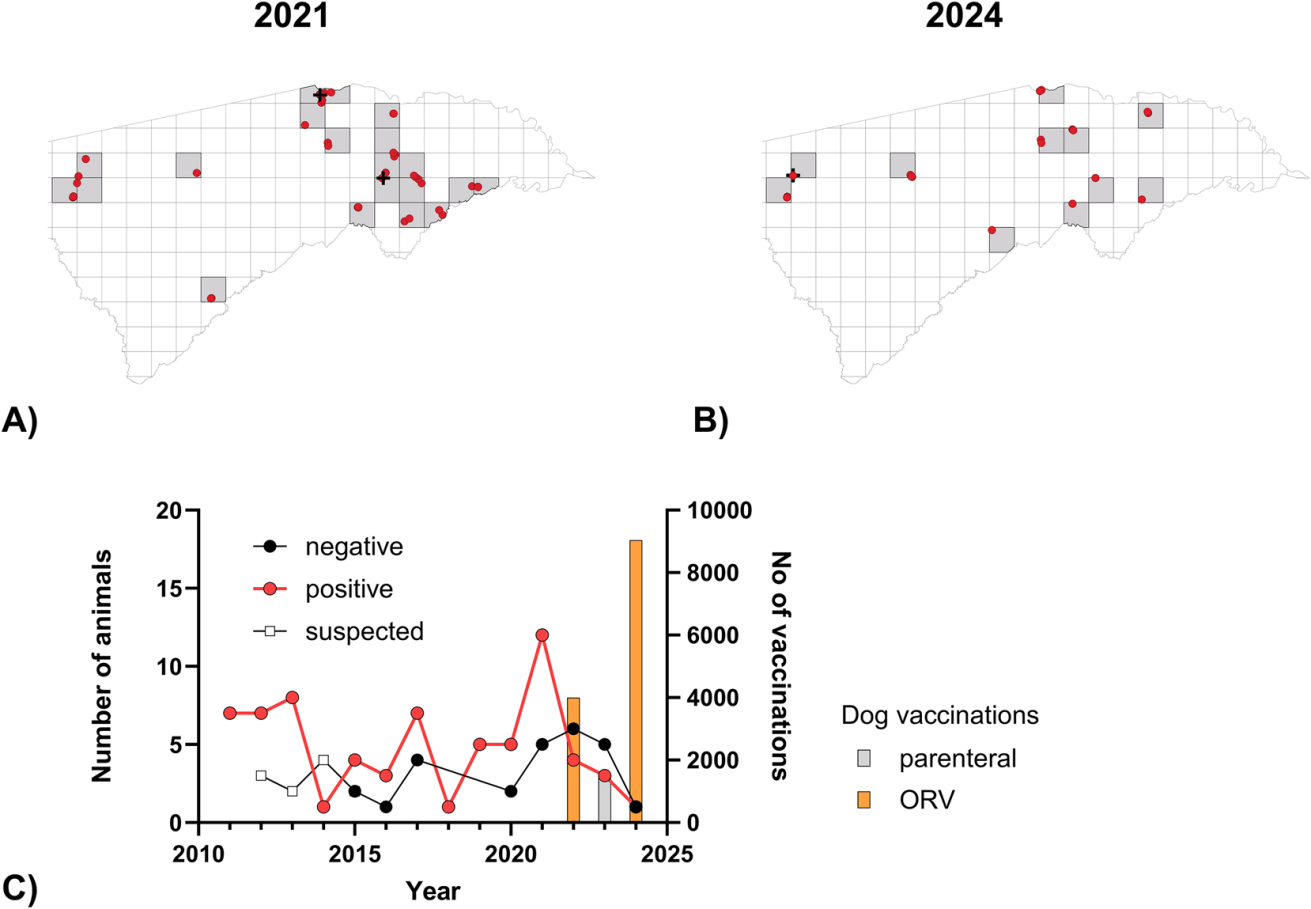
Maps of the Zambezi region, overlaid with a 10 × 10 km grid and point data from the KAP study in 2021 (**A**) and the post vaccination monitoring study in 2024 (**B**), where respondents indicated the presence of rabies in animals during the previous year. The dots in the grid cells represent the location of respondents who answered positively to the question about the presence of rabies. Human rabies (black cross) is indicated. The maps were created using QGIS (version 3.40.2; QGIS Development Team, 2024; https://qgis.org/). (**C**) Surveillance data from the Zambezi region over the years and number of dog vaccinations shown.

Respondents were also asked about the presence of rabies in animals and humans. In 2024, 4.6% of the respondents reported rabies cases in their neighborhood (11 grid cells affected), compared to 12% in 2021, when 20 grid cells were affected. Eight of the 11 grid cells for 2024 were also affected in 2021 (Fig. 6). Two cases of human rabies were reported in 2021, while four were reported in 2024, with three of these reports coming from a single village within a 200 m radius, suggesting a single rabies source.

Regarding dog bites, 61 respondents reported incidents involving household members. With a surveyed population of 2,523, this equates to an annual dog bite incidence of 2,418 per 100,000 people, more than double the 2021 incidence (Supplementary Fig. 4). Most bites (67.2%) were inflicted by (owned) pet dogs, followed by stray dogs (16.4%). Four people were bitten by orally vaccinated dogs within six hours after vaccination, and three of them received post-exposure prophylaxis (PEP), as recommended in the safety guidelines. Overall, PEP was administered in 78.7% of dog bite cases.

## Discussion

Vaccination of dogs is the proven way to control and eventually eliminate rabies in the dog population. The annual coverage needed to confer herd immunity was modelled to be above 70%^17^, but this threshold often represents a programmatic target rather than a proven guarantee in every context^18–20^. The results from the 2024 oral rabies vaccination (ORV) campaign in the Zambezi region of Namibia indicate that the campaign achieved a significant level of coverage and provided useful insights into the effectiveness of rabies control measures. The total number of baits offered, both directly to dogs and indirectly via dog owners, reflects an organized and widespread vaccination effort. Within this campaign, teams managed to deliver a substantial number of baits within the planned four-day period (Figs. 2 and 3). This is very encouraging as the area is difficult to cover, even with 4WD trucks. The Zambezi Region is one of the most flood-prone areas in southern Africa, shaped by seasonal rainfall, river overflows from the Zambezi and Chobe, and extensive marshlands. These environmental dynamics profoundly affect accessibility, and therefore the effectiveness and feasibility of oral rabies vaccination interventions. For these reasons, we chose the dry season (June) as the most strategic and effective window for conducting ORV.

**Fig. 7:**
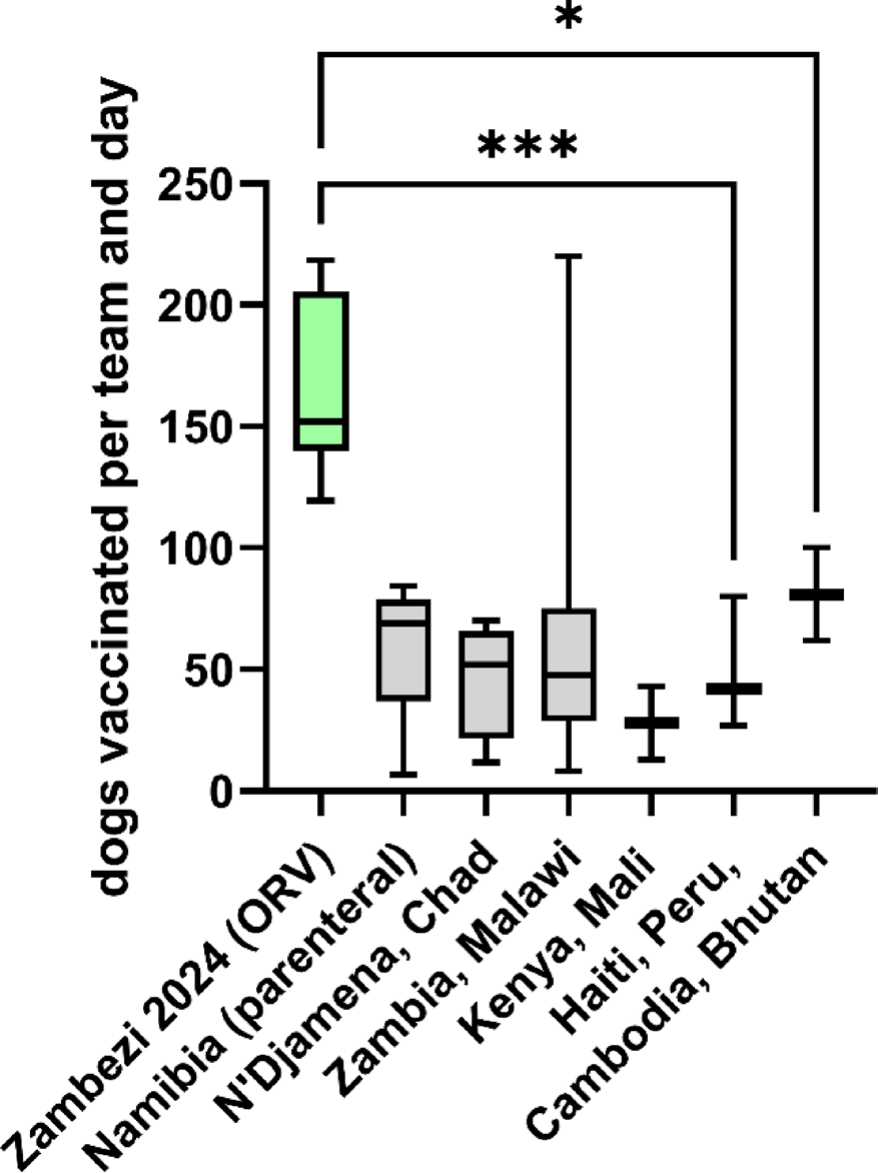
Boxplot-chart of team performance for dog vaccinations, with whiskers indicating the range. One-way ANOVA followed by Dunnett’s multiple comparisons test (GraphPad Prism version 10.4.0 for Windows, GraphPad Software, San Diego, California USA, http://www.graphpad.com) demonstrated significant differences in the mean of the ORV campaign (green) compared to all other means. Data was derived from published studies^3,4,14,22–28,30–36^ and summarized per country, region or continent (raw data, see Supplementary Table S4).

The average number of baits directly distributed per team per hour (25.5) and per team per day (164) suggests a high operational efficiency especially considering the fact that the approach can be considered door-to-door (D2D) vaccination (Fig. 7). The mean of dogs vaccinated per team and hour was significantly higher than that of published data from parenteral vaccination campaigns. For Namibia, during the ORV campaign in 2024 the number of vaccinated dogs per team and day was more than three times higher than that of the mass dog vaccination campaign in 2024 in the NCA (*N* = 53) and five times that of the campaigns in 2018 (*N* = 33). In other settings, vaccination effectiveness was reported or estimated as 28–43 dogs/team/day for Nairobi, Kenya^21^, 27–42 for Haiti^22^, 62 for Cambodia^23^, 100 for N’Djamena, Chad^24,25^, 100 for Bhutan^26^, 143 for Lusaka^27^ and 220 for Blantyre, Malawi^14^. However, there are only a few studies published that report on vaccination effectiveness in rural areas. In rural Zambia, during parenteral central-point campaigns 71 dogs^27^ and between 8 and 10 dogs were vaccinated per team and day^28^, whereas in rural Malawi the range was 37–73, depending on whether D2D vaccination or a static point was used^29^.

To this end, in our campaign owner distribution of baits was very helpful as dogs were sometimes at different homesteads several kilometers within bushland, making it extremely difficult and time consuming to reach them directly. With the inclusion of these baits, teams vaccinated on average 183 dogs per day.

One of the key takeaways from this study is that upscaling of the campaign from four to ten teams and from 4,000 to 10,000 baits, respectively, did not decrease the effectiveness, thus even large-scale campaigns are feasible. The self-initiated additional baiting efforts on Impalila Island and the follow-up distribution by DVS in underserved areas further demonstrate the commitment of the responsible authorities to achieving comprehensive rabies vaccination coverage.

Several methods can be used to estimate vaccination coverage including (i) the use of pre-campaign estimates of dog population size through human-to-dog ratios (HDRs) as the denominator^37^, and the number of dogs vaccinated during the campaign as the numerator^25,38–40^, (ii) sight-resight count surveys of vaccinated marked dogs^41,42^ and (iii) post-vaccination household surveys to estimate the proportion of vaccinated dogs^37,43^. While serology can be used to assess the immune response to vaccination in a vaccinated individual^44^, sero-epidemiological studies are not recommended for post-vaccination monitoring as antibody levels are variable and potentially rapidly decline^1^. Also, with the target of free-roaming hard-to-reach dogs a subsequent sampling of these animals is inherently difficult and prone to bias.

Therefore, for the first time in Namibia, we have used an additional post-vaccination household survey to compare and confirm vaccination rates.

The calculated vaccination coverage of 47.9% for the Zambezi region based on the estimated dog population (estimated from HDR) aligns with the expected outcomes of an ORV campaign in a region where logistical and environmental challenges exist. In fact, this concerted short-term intervention was more effective than previous campaigns^4^ and could increase the dog vaccination coverage to similar levels as in the Serengeti district of Tanzania, where annual mass dog vaccinations had been implemented before^43^.

It is important to note that the vaccination coverage in the Zambezi region was further increased to 56.8% when accounting for the previous mass dog vaccination effort. Interestingly, the rise in vaccination rates is also reflected in the post vaccination survey, where 59.4% of households and 54.5% of dogs were declared as vaccinated in 2024, a tremendous increase (by over 35%) compared to the survey in 2021. This was achieved in just the same amount of time as in 2022^8^, but with twice as many vaccination teams. The coverage data, analyzed at the level of 10 × 10 km grid cells (Fig. 4), reveals that a substantial portion of the region received adequate vaccination coverage, with an average of 60% coverage across all grid cells. However, the presence of a few grid cells with vaccination coverage below 20%—particularly those with populations exceeding 500—suggests the need for targeted follow-up in these areas. The more detailed spatial analysis of vaccination points, buffered by a 500-meter radius, indicates that a large proportion of the region’s human population (81%) and buildings (73%) were covered by the vaccination campaign (Supplementary Fig. 2). This spatial overlap underscores the potential of ORV campaigns to target dogs directly in their environment, with limited gaps. The use of building footprints and human population density data strengthens the spatial analysis^45^, providing a more accurate representation of coverage and highlighting areas where additional outreach may be required.

While ORV as a tool was generally very well accepted, barriers to vaccination still exist as the post-vaccination survey showed. A substantial proportion of respondents (70%) cited lack of awareness as the primary reason for not vaccinating their dogs, which highlights the need for continued education and outreach efforts. While the percentage of households citing age and other factors (such as distance or handling issues) has decreased compared to the initial KAP study in 2021^11^, these factors may still play a role in limiting vaccination coverage in some areas. Generally, the wrong perception that puppies cannot be vaccinated against rabies is widespread, as other surveys in Tanzania^46^, Sri Lanka^29^ and the Philippines^47^ revealed similar results.

One key question to be answered is whether increased vaccination efforts ultimately lead to an effective rabies control. The presence of animal rabies in 2024 (Fig. 6) demonstrates that the performed campaigns had not resulted in a complete stop of transmission. In fact, this was not expected or envisaged, as the two ORV interventions had the character of proof-of-principle field trials with the goal to find ways for optimization of this approach under large field settings. It was evident that vaccination coverages, particularly in the first one, would be well below the threshold of 70% at which rabies transmission is likely to be stopped^48^. However, the lower reported presence of rabies in animals in 2024 (4.6%) compared to 2021 (12%) is a positive sign, suggesting that vaccination efforts even half a year apart (Fig. 6) were having an impact on controlling the disease. Similar effects were also observed in rural Tanzania^37^, where some of the foundations for dog rabies control in Africa were laid. It can be assumed that if several campaigns with the same effectiveness as in 2024 were carried out at shorter intervals, rabies would quickly disappear in the Zambesi region.

Despite this positive trend, the presence of human rabies cases and the high rate of dog bites (2,418 per 100,000 people) remain a concern. Although the majority of dog bites in the Zambesi region were inflicted by pet dogs, the fact that 78.7% of bite victims received appropriate post-exposure prophylaxis (PEP) is reassuring, demonstrating a good level of adherence to safety protocols. Notably, the safety instructions for ORV use were followed^9^ and most persons bitten immediately after dogs had received a vaccine bait were given PEP. Albeit the vaccine is safe^49^, offering PEP follows the precautionary principle as vaccine virus can be present for several hours in the saliva of a recently orally vaccinated dog^50^.

There are certain limitations to this study or to the circumstances of rabies surveillance and control. First, due to budget constraints, the number of baits was fixed to 10,000 and therefore it was inevitable that the vaccination coverage would be below 70%. The increase in dog ownership in the surveyed population from 47.1% to 70.2% and the reduced HDR from 5.7 to 3.8 needs careful interpretation. As such change over a short period of time is unlikely and the differences are unprecedented in the NCAs, this could point to a bias of selections towards dog-owning households. Also, there may have been a slight overestimation of vaccination rates as self-reported data can be positively biased by owners, particularly if vaccination was compulsory and seen as socially desirable^51^, and the survey was performed by official DVS staff. Human rabies cases reported in 2024 (*N* = 4) were again followed up by DVS and it seems that there was only one human rabies case at that particular village in 2024 and households were referring to that same person as a relative who succumbed to rabies. Evidently, the question was not phrased or explained correctly as to circumvent misinterpretations.

Another limitation is the use of the human population census as proxy for dog population and hence vaccination coverage. Only recently, new census data became available, indicating a population growth to 142.373 people in the Zambezi region^52^, an increase of 34% compared to the adjusted census data from 2011 of 106.000^15^. It is difficult to say how exactly this population growth affected the dog population in the area, however, this population growth in numbers and spatial extension has to be accounted for in any planning activities, especially in low- and middle-income countries (LMICs).

The ultimate proof for an effective campaign is the reduction of rabies incidence both in animals and humans. To this end, however, even though the laboratory-based rabies surveillance in Namibia is exemplary^53^, the level of submissions for rabies from the Zambesi region was too low to infer changes in incidence. One main constraint observed during a previous KAP study was the lacking willingness to submit samples for investigation^11^. In fact, also in 2024 data from the Zambezi region showed that only one of 13 dogs that bit and were subsequently killed was submitted for laboratory testing. To overcome the limitations of laboratory-based surveillance, in this study survey-based surveillance was additionally used and although this has inherent limitations as no laboratory confirmation is included, it was more capable of monitoring disease trends, both in humans and animals indicating an effect of vaccination on the occurrence of rabies. However, it was shown before in Tanzania that household surveys are time-consuming and costly^43^, and cannot replace a functioning surveillance system.

## Conclusions

The 2024 ORV campaign in the Zambezi region establishes a robust foundation for future rabies control initiatives, demonstrating the feasibility of large-scale, spatially informed vaccination campaigns, and underscoring the importance of sustained, coordinated efforts across different sectors to combat rabies and protect both animals and human communities. Both HDR and GIS-derived vaccination coverages calculated from the number of vaccinated animals seem to represent the local coverages, thus making costly post-vaccination surveys dispensable.

In terms of future directions, several areas for improvement and expansion emerge. First, increasing the use of ORV and owner-distributed baits, particularly in regions with hard-to-reach dog populations, can enhance both cost-effectiveness and vaccination coverage. Indeed, in a recent opinion paper on rabies control in Africa, ORV as an innovative tool is explicitly mentioned as one requirement for reaching the goal of rabies control^54^.

Furthermore, promptly identifying areas with low coverage following rabies vaccination campaigns would enable more precise and impactful targeted interventions. Lastly, continued efforts are essential to raise awareness about the importance of dog rabies vaccination and to encourage the submission of rabies-suspected animals to the Department of Veterinary Services (DVS).

## Supplementary Information

Below is the link to the electronic supplementary material.

Supplementary Material 1

## Data Availability

The data that support the findings of this study are openly available in Zenodo at https://zenodo.org/, reference number 10.5281/zenodo.14744857.
