# Supplementary material for: Evaluating the 2024 dog oral rabies vaccination campaign in the Zambezi region, Namibia using GIS and household surveys
Source: Sci Rep. 2026 Mar 16;16:9204. doi: 10.1038/s41598-026-38405-x (PMC12996389; doi:10.1038/s41598-026-38405-x)
Supplement: Supplementary file 1 — Supplementary Material 1 [file 41598_2026_38405_MOESM1_ESM.docx]

**Evaluating the 2024 Dog Oral Rabies Vaccination Campaign in the Zambezi Region, Namibia Using GIS and Household Surveys**

Conrad M. Freuling^1#^, Mainelo Beatrice Shikongo^2^, Frank Busch^3^, Sarah Gottlieb^4^, Reinhold Haimbodi^5^, Naindji Haindongo^5^, Chantal Hansen^6^, Juliet Kabajani^6^, Joseph Kapapero^5^, Muesee Kasaona^6^, Mattia Marconcini^7^, Jeremia Namusheshe^2^, Nzwana Silume^2^, Tenzin Tenzin^8^, Ad Vos^9^, Thomas Müller^1^

^1^ Institute of Molecular Virology and Cell Biology, Friedrich-Loeffler-Institut (FLI), WOAH Reference Laboratory for Rabies, Greifswald-Insel Riems, Germany

^2^State Veterinary Office, Ministry of Agriculture, Water & Land Reform, Directorate of Veterinary Services, Zambezi region, Katima Mulilo, Namibia.

^3^Institute of International Animal Health/One Health, Friedrich-Loeffler-Institut (FLI), Greifswald-Insel Riems, Germany

^4^State Veterinary Office, Ministry of Agriculture, Water & Land Reform, Directorate of Veterinary Services, Kavango East region, Nkurunkuru, Namibia.

^5^Ministry of Agriculture, Water and Land Reform, Directorate of Veterinary Services, Windhoek, Namibia

^6^Central Veterinary Laboratory (CVL), Ministry of Agriculture, Water and Land Reform, Directorate of Veterinary Services, Windhoek, Namibia

^7^German Aerospace Center – DLR, Cologne, Germany

^8^World Organisation for Animal Health (WOAH), Sub-Regional Representation for Southern Africa, Gaborone, Botswana

^9^CEVA Sante Animale, Libourne, France

# corresponding author

**Supplementary information**

**Figure S1:** Map of the study area and team areas shaded in different colours. Human settlement features (black dots) is based on High Resolution Settlement Layer (HRSL) from Meta (formerly Facebook). The map was created using QGIS (version 3.40.2; QGIS Development Team, 2024; https://qgis.org/) with OpenStreetMap data as the basemap (© OpenStreetMap contributors; https://www.openstreetmap.org).


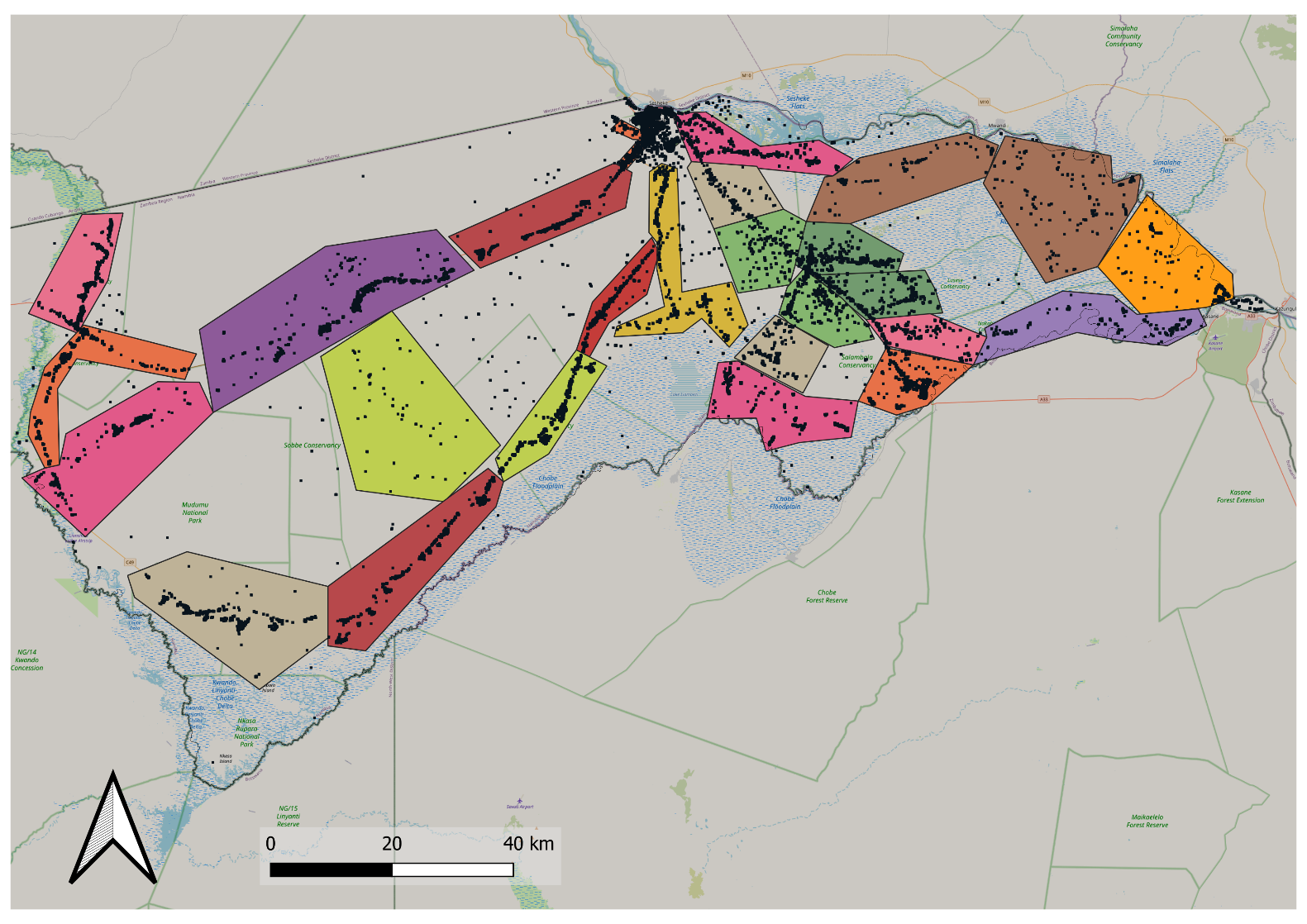


**Figure S2:** Map of the study area showing 500 m buffer zones around each vaccinated dog. Human settlement features within the buffer zones are indicated by black dots and were derived from the Google Buildings layer (https://sites.research.google/gr/open-buildings/). The map was created using QGIS (version 3.40.2; QGIS Development Team, 2024; https://qgis.org/) with OpenStreetMap data as the basemap (© OpenStreetMap contributors; <https://www.openstreetmap.org>).

).
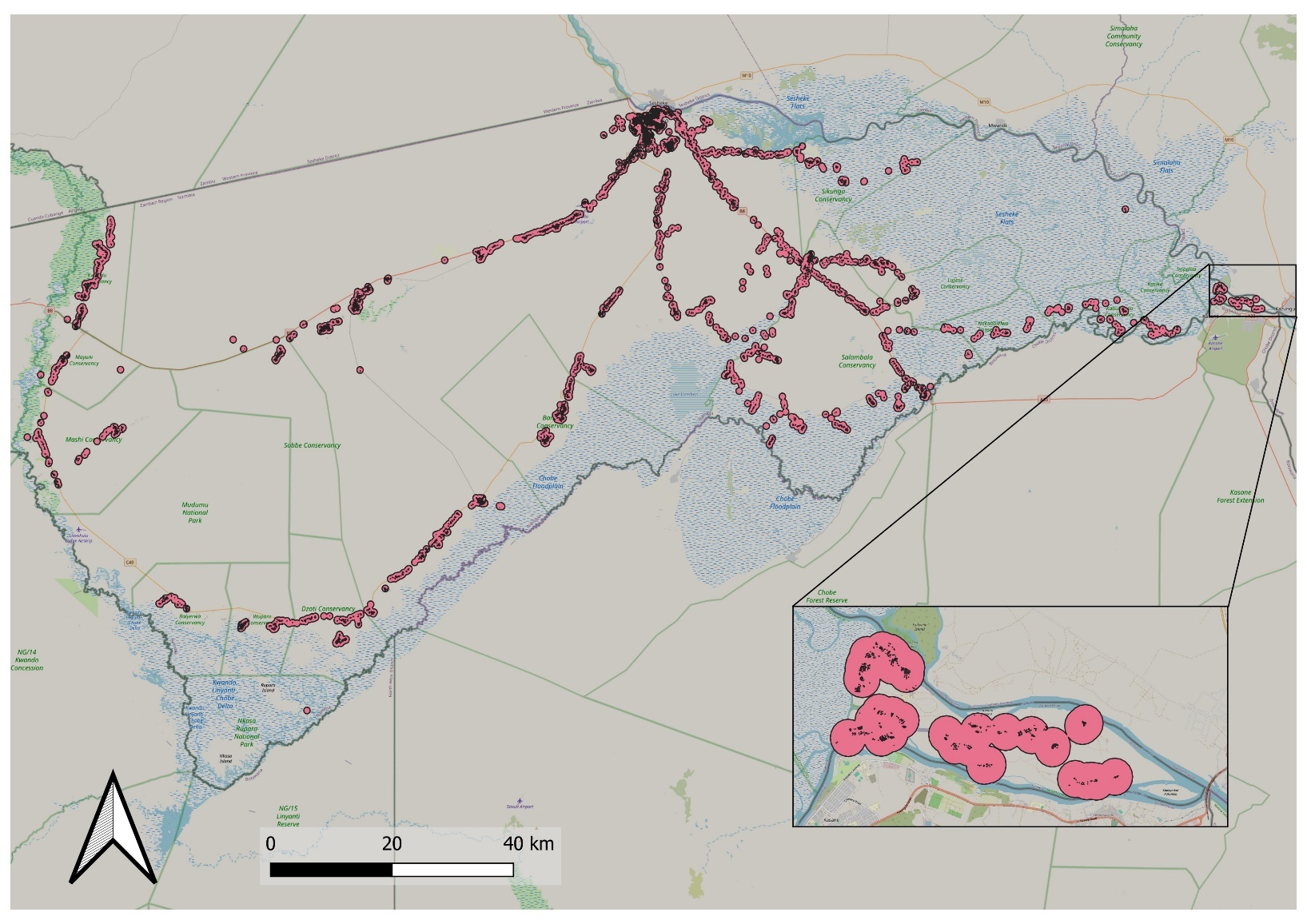


**Figure S3:** Map of the study area with individual locations for the post vaccination survey indicated (red dots). The map was created using QGIS (version 3.40.2; QGIS Development Team, 2024; https://qgis.org/) with OpenStreetMap data as the basemap (© OpenStreetMap contributors; https://www.openstreetmap.org).


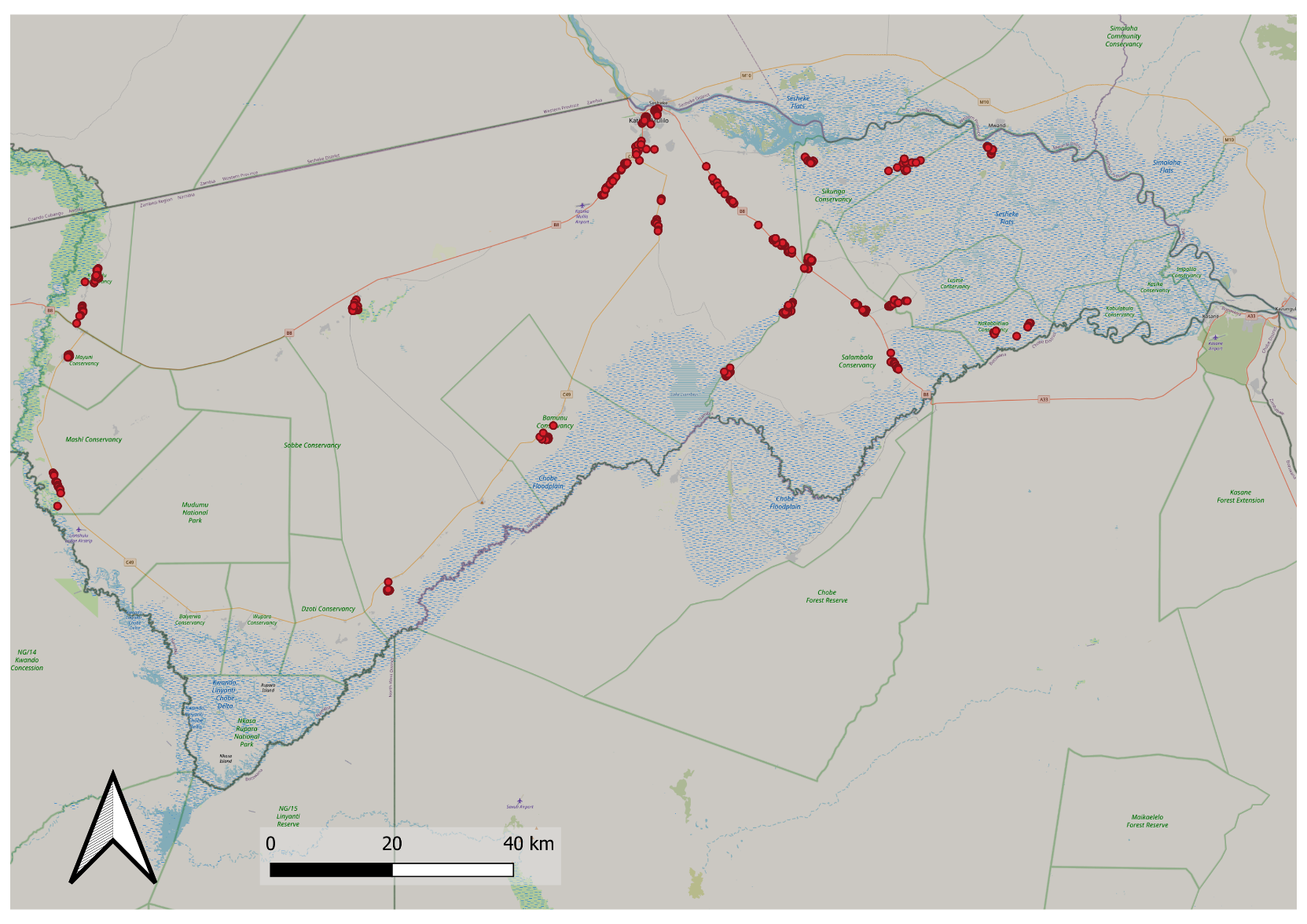


**Figure S4:** Dog bite incidence (cases per 100,000 population per year) by region. Points represent estimated incidence rates, with vertical error bars indicating 95% confidence intervals. The dashed horizontal line indicates the national mean incidence, with the shaded band representing the corresponding confidence interval. The data is derived from the KAP study in 2021 (Tenzin et al., 2024, DOI: 10.1371/journal.pntd.0011631). Zambezi (2024) denotes the most recent estimate for that region from this post-vaccination survey.


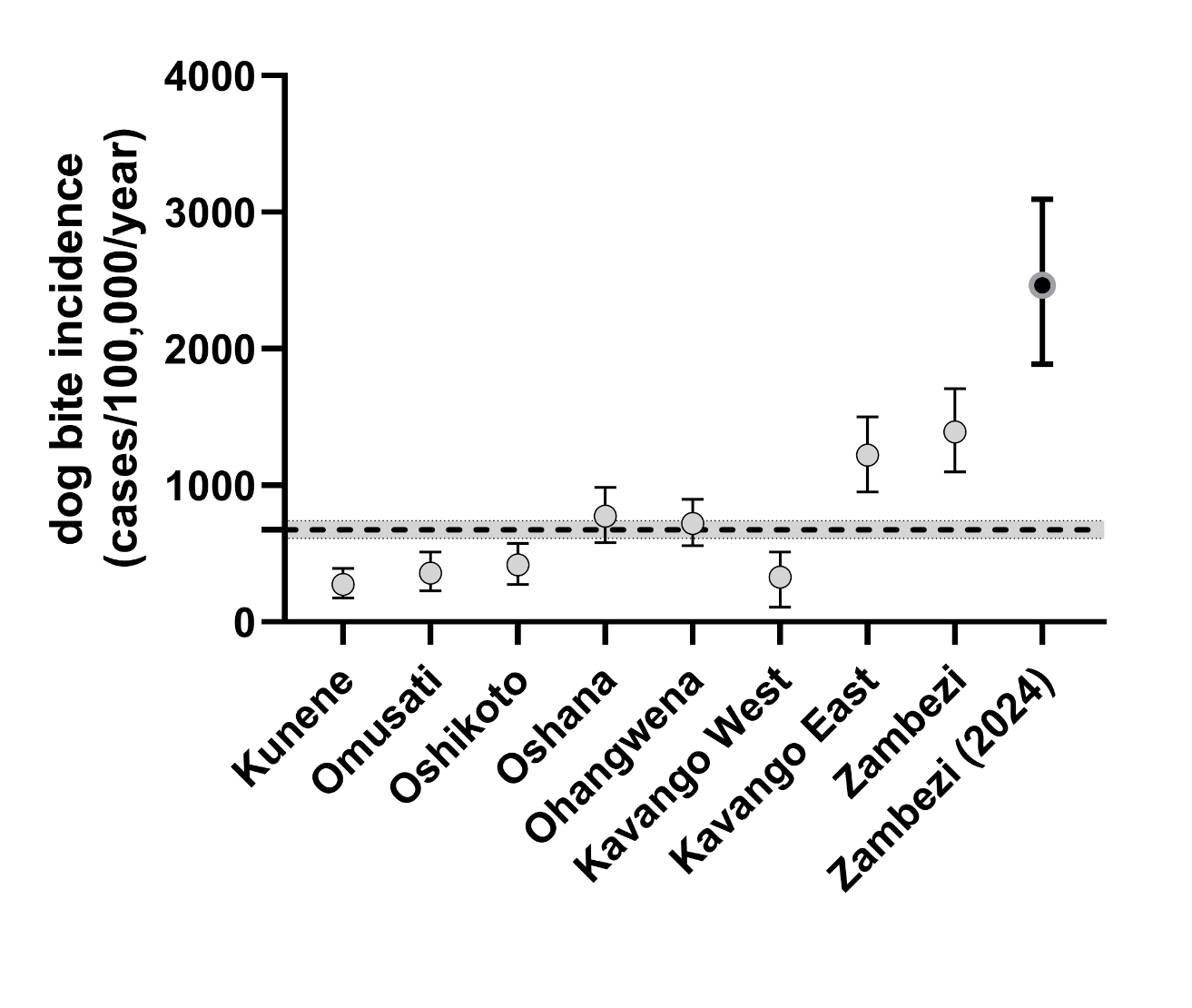


**Table S1:** Questionaire used for the post vaccination survey as implemented in the WVS data collection app (<https://missionrabies.com/app/>).

| Question Text | Question Value | Control Type | Compulsory | Option Text | Option Value | Sub-Options |
| --- | --- | --- | --- | --- | --- | --- |
| Please remind the respondent that the questionnaire is anonymous and that their names are not recorded. Their answers are confidential and will not be linked to them. The results of this survey may be published in a scientific journal. | NA | Instruction | No | NA | NA | NA |
| Do you consent to participate in this survey? | consent | Radio Button | Yes | Yes | yes | NA |
| Do you consent to participate in this survey? | consent | Radio Button | Yes | No | no | NA |
| Type of Settlement | settlement_type | Radio Button | Yes | Village/Rural | rural | NA |
| Type of Settlement | settlement_type | Radio Button | Yes | Town/Urban | urban | NA |
| What is your level of education? | education_level | Radio Button | Yes | I did not attend any school | no_school | NA |
| What is your level of education? | education_level | Radio Button | Yes | Primary level | primary_level | NA |
| What is your level of education? | education_level | Radio Button | Yes | Secondary level | secondary_level | NA |
| What is your level of education? | education_level | Radio Button | Yes | Graduate | graduate | NA |
| What is your level of education? | education_level | Radio Button | Yes | Other (please specify) | other | NA |
| What is your profession? | profession | Radio Button | Yes | Farmer | farmer | NA |
| What is your profession? | profession | Radio Button | Yes | Business Owner | business | NA |
| What is your profession? | profession | Radio Button | Yes | Government Employee | government | NA |
| What is your profession? | profession | Radio Button | Yes | Private Enterprise/Corporate Employee | employee | NA |
| What is your profession? | profession | Radio Button | Yes | Student | student | NA |
| What is your profession? | profession | Radio Button | Yes | No profession | none | NA |
| What is your profession? | profession | Radio Button | Yes | Other | other | NA |
| Do you own any livestock? | livestock_ownership | Radio Button | Yes | Yes | yes | NA |
| Do you own any livestock? | livestock_ownership | Radio Button | Yes | No | no | NA |
| How many people live your household? | household_size | Textbox | Yes | NA | NA | NA |
| Do you own any dogs? | dog_ownership | Radio Button | Yes | Yes | yes | NA |
| Do you own any dogs? | dog_ownership | Radio Button | Yes | No | no | NA |
| How many dogs do you own? | number_dogs | Numeric | Yes | NA | NA | NA |
| Did your dogs get vaccinated during 2023 or 2024? | vaccination_status | Radio Button | Yes | Yes | yes | NA |
| Did your dogs get vaccinated during 2023 or 2024? | vaccination_status | Radio Button | Yes | No | no | NA |
| How many of your dogs were vaccinated? | dogs_vaccinated | Numeric | Yes | NA | NA | NA |
| What were the reasons for not vaccinating? | reason_no_vaccination | Multi Select | Yes | Not aware of vaccination campaign | awareness | NA |
| What were the reasons for not vaccinating? | reason_no_vaccination | Multi Select | Yes | Vaccination points too far away from my house | distance | NA |
| What were the reasons for not vaccinating? | reason_no_vaccination | Multi Select | Yes | Dog was aggressive & could not be caught/handled | handling | NA |
| What were the reasons for not vaccinating? | reason_no_vaccination | Multi Select | Yes | Dog already vaccinated at the veterinary clinic | already_vaccinated | NA |
| What were the reasons for not vaccinating? | reason_no_vaccination | Multi Select | Yes | Dog too young (puppy) | too_young | NA |
| What were the reasons for not vaccinating? | reason_no_vaccination | Multi Select | Yes | Dog too old | too_old | NA |
| What were the reasons for not vaccinating? | reason_no_vaccination | Multi Select | Yes | Dog pregnant | pregnant | NA |
| What were the reasons for not vaccinating? | reason_no_vaccination | Multi Select | Yes | Dog lactating | lactating | NA |
| What were the reasons for not vaccinating? | reason_no_vaccination | Multi Select | Yes | Dog was sick | sickness | NA |
| What were the reasons for not vaccinating? | reason_no_vaccination | Multi Select | Yes | No time to take dog for vaccination | no_time | NA |
| What were the reasons for not vaccinating? | reason_no_vaccination | Multi Select | Yes | Other reasons (please specify) | other | NA |
| How were your dogs vaccinated? | vaccination_provider | Multi Select | Yes | During an oral vaccination campaign in 2024 | ORV | NA |
| How were your dogs vaccinated? | vaccination_provider | Multi Select | Yes | Parenterally | parenterally | NA |
| How were your dogs vaccinated? | vaccination_provider | Multi Select | Yes | At vaccination point during targeted campaign | campaign | NA |
| How were your dogs vaccinated? | vaccination_provider | Multi Select | Yes | At cattle crush pen during cattle vaccination | crush_pen | NA |
| How were your dogs vaccinated? | vaccination_provider | Multi Select | Yes | At the veterinary clinic/extension centres | extension_centres | NA |
| How were your dogs vaccinated? | vaccination_provider | Multi Select | Yes | At a private veterinary clinic | private_vet | NA |
| How were your dogs vaccinated? | vaccination_provider | Multi Select | Yes | Other (Please specify) | other | NA |
| How were your dogs vaccinated? | vaccination_provider | Multi Select | Yes | I don't remember | no_data | NA |
| Who brought the dogs to vaccination? | attendant_clinic | Radio Button | Yes | Children | children | NA |
| Who brought the dogs to vaccination? | attendant_clinic | Radio Button | Yes | Adult family member | adult | NA |
| Who brought the dogs to vaccination? | attendant_clinic | Radio Button | Yes | Both | both | NA |
| Who brought the dogs to vaccination? | attendant_clinic | Radio Button | Yes | Not applicable (ORV) | not_appl | NA |
| How far did you have to travel for the vaccination? | travel_distance | Radio Button | Yes | 1km | 1km | NA |
| How far did you have to travel for the vaccination? | travel_distance | Radio Button | Yes | 2km | 2km | NA |
| How far did you have to travel for the vaccination? | travel_distance | Radio Button | Yes | 3km | 3km | NA |
| How far did you have to travel for the vaccination? | travel_distance | Radio Button | Yes | 4km | 4km | NA |
| How far did you have to travel for the vaccination? | travel_distance | Radio Button | Yes | More than 4km | more_4km | NA |
| How far did you have to travel for the vaccination? | travel_distance | Radio Button | Yes | I don't know | no_data | NA |
| How far did you have to travel for the vaccination? | travel_distance | Radio Button | Yes | Less than 500m | under_500m | NA |
| How far did you have to travel for the vaccination? | travel_distance | Radio Button | Yes | 1km | 1km | NA |
| How long does it take you to bring your dog for vaccination, vaccinate and return home? | travel_time | Radio Button | Yes | 1 hour | 1hr | NA |
| How long does it take you to bring your dog for vaccination, vaccinate and return home? | travel_time | Radio Button | Yes | 2 hours | 2hrs | NA |
| How long does it take you to bring your dog for vaccination, vaccinate and return home? | travel_time | Radio Button | Yes | 3 hours | 3hrs | NA |
| How long does it take you to bring your dog for vaccination, vaccinate and return home? | travel_time | Radio Button | Yes | More than 3 hours | more_3hrs | NA |
| How long does it take you to bring your dog for vaccination, vaccinate and return home? | travel_time | Radio Button | Yes | 1/2 hour | 05_hr | NA |
| How long does it take you to bring your dog for vaccination, vaccinate and return home? | travel_time | Radio Button | Yes | 1/4 hour | 025_hr | NA |
| How long does it take you to bring your dog for vaccination, vaccinate and return home? | travel_time | Radio Button | Yes | less than 15 min | less_025hr | NA |
| Where did you learn about rabies vaccinations? | sensitization | Multi Select | Yes | Through vets/extension staff | vet | NA |
| Where did you learn about rabies vaccinations? | sensitization | Multi Select | Yes | Through traditional leaders | tradional_authority | NA |
| Where did you learn about rabies vaccinations? | sensitization | Multi Select | Yes | Via radio | radio | NA |
| Where did you learn about rabies vaccinations? | sensitization | Multi Select | Yes | From my children | children | NA |
| Where did you learn about rabies vaccinations? | sensitization | Multi Select | Yes | Other (please specify) | other | NA |
| Where did you learn about rabies vaccinations? | sensitization | Multi Select | Yes | Via TV | tv | NA |
| Do you think vaccination with an oral bait is a good tool? | ORV_tool | Radio Button | Yes | strongly disagree | 1 | NA |
| Do you think vaccination with an oral bait is a good tool? | ORV_tool | Radio Button | Yes | disagree | 2 | NA |
| Do you think vaccination with an oral bait is a good tool? | ORV_tool | Radio Button | Yes | neutral | 3 | NA |
| Do you think vaccination with an oral bait is a good tool? | ORV_tool | Radio Button | Yes | agree | 4 | NA |
| Do you think vaccination with an oral bait is a good tool? | ORV_tool | Radio Button | Yes | strongly agree | 5 | NA |
| Do you think vaccination with an oral bait is a good tool? | ORV_tool | Radio Button | Yes | Children | children | NA |
| Do you think vaccination with an oral bait is a good tool? | ORV_tool | Radio Button | Yes | Adult family member | adult | NA |
| Do you think vaccination with an oral bait is a good tool? | ORV_tool | Radio Button | Yes | Both | both | NA |
| My dogs could only be vaccinated by oral baits | ORV_dog_vaccination | Radio Button | Yes | strongly disagree | 1 | NA |
| My dogs could only be vaccinated by oral baits | ORV_dog_vaccination | Radio Button | Yes | disagree | 2 | NA |
| My dogs could only be vaccinated by oral baits | ORV_dog_vaccination | Radio Button | Yes | neutral | 3 | NA |
| My dogs could only be vaccinated by oral baits | ORV_dog_vaccination | Radio Button | Yes | agree | 4 | NA |
| My dogs could only be vaccinated by oral baits | ORV_dog_vaccination | Radio Button | Yes | strongly agree | 5 | NA |
| My dogs could only be vaccinated by oral baits | ORV_dog_vaccination | Radio Button | Yes | Children | children | NA |
| My dogs could only be vaccinated by oral baits | ORV_dog_vaccination | Radio Button | Yes | Adult family member | adult | NA |
| My dogs could only be vaccinated by oral baits | ORV_dog_vaccination | Radio Button | Yes | Both | both | NA |
| The best way is to combine parenteral vaccination of puppies with oral vaccination of free roaming dogs | ORV_combination_vaccination | Radio Button | Yes | strongly disagree | 1 | NA |
| The best way is to combine parenteral vaccination of puppies with oral vaccination of free roaming dogs | ORV_combination_vaccination | Radio Button | Yes | disagree | 2 | NA |
| The best way is to combine parenteral vaccination of puppies with oral vaccination of free roaming dogs | ORV_combination_vaccination | Radio Button | Yes | neutral | 3 | NA |
| The best way is to combine parenteral vaccination of puppies with oral vaccination of free roaming dogs | ORV_combination_vaccination | Radio Button | Yes | agree | 4 | NA |
| The best way is to combine parenteral vaccination of puppies with oral vaccination of free roaming dogs | ORV_combination_vaccination | Radio Button | Yes | strongly agree | 5 | NA |
| The best way is to combine parenteral vaccination of puppies with oral vaccination of free roaming dogs | ORV_combination_vaccination | Radio Button | Yes | Children | children | NA |
| The best way is to combine parenteral vaccination of puppies with oral vaccination of free roaming dogs | ORV_combination_vaccination | Radio Button | Yes | Adult family member | adult | NA |
| The best way is to combine parenteral vaccination of puppies with oral vaccination of free roaming dogs | ORV_combination_vaccination | Radio Button | Yes | Both | both | NA |
| Did you observe any negative effects in your orally vaccinated dog? | ORV_negative_effect | Radio Button | Yes | yes | 1 | NA |
| Did you observe any negative effects in your orally vaccinated dog? | ORV_negative_effect | Radio Button | Yes | no | 0 | NA |
| Did you observe any negative effects in your orally vaccinated dog? | ORV_negative_effect | Radio Button | Yes | strongly disagree | 1 | NA |
| Did you observe any negative effects in your orally vaccinated dog? | ORV_negative_effect | Radio Button | Yes | disagree | 2 | NA |
| Did you observe any negative effects in your orally vaccinated dog? | ORV_negative_effect | Radio Button | Yes | neutral | 3 | NA |
| Did you observe any negative effects in your orally vaccinated dog? | ORV_negative_effect | Radio Button | Yes | agree | 4 | NA |
| Did you observe any negative effects in your orally vaccinated dog? | ORV_negative_effect | Radio Button | Yes | strongly agree | 5 | NA |
| Did you observe any negative effects in your orally vaccinated dog? | ORV_negative_effect | Radio Button | Yes | Children | children | NA |
| Did you observe any negative effects in your orally vaccinated dog? | ORV_negative_effect | Radio Button | Yes | Adult family member | adult | NA |
| Did you observe any negative effects in your orally vaccinated dog? | ORV_negative_effect | Radio Button | Yes | Both | both | NA |
| Oral rabies vaccination schould be included in the vaccination strategy. | ORV_strategy | Radio Button | Yes | strongly disagree | 1 | NA |
| Oral rabies vaccination schould be included in the vaccination strategy. | ORV_strategy | Radio Button | Yes | disagree | 2 | NA |
| Oral rabies vaccination schould be included in the vaccination strategy. | ORV_strategy | Radio Button | Yes | neutral | 3 | NA |
| Oral rabies vaccination schould be included in the vaccination strategy. | ORV_strategy | Radio Button | Yes | agree | 4 | NA |
| Oral rabies vaccination schould be included in the vaccination strategy. | ORV_strategy | Radio Button | Yes | strongly agree | 5 | NA |
| Oral rabies vaccination schould be included in the vaccination strategy. | ORV_strategy | Radio Button | Yes | Children | children | NA |
| Oral rabies vaccination schould be included in the vaccination strategy. | ORV_strategy | Radio Button | Yes | Adult family member | adult | NA |
| Oral rabies vaccination schould be included in the vaccination strategy. | ORV_strategy | Radio Button | Yes | Both | both | NA |
| I would also pick up baits from a central source (store, headman, etc) to vaccinate my own dogs. | ORV_pickup_baits | Radio Button | Yes | strongly disagree | 1 | NA |
| I would also pick up baits from a central source (store, headman, etc) to vaccinate my own dogs. | ORV_pickup_baits | Radio Button | Yes | disagree | 2 | NA |
| I would also pick up baits from a central source (store, headman, etc) to vaccinate my own dogs. | ORV_pickup_baits | Radio Button | Yes | neutral | 3 | NA |
| I would also pick up baits from a central source (store, headman, etc) to vaccinate my own dogs. | ORV_pickup_baits | Radio Button | Yes | agree | 4 | NA |
| I would also pick up baits from a central source (store, headman, etc) to vaccinate my own dogs. | ORV_pickup_baits | Radio Button | Yes | strongly agree | 5 | NA |
| I would also pick up baits from a central source (store, headman, etc) to vaccinate my own dogs. | ORV_pickup_baits | Radio Button | Yes | Children | children | NA |
| I would also pick up baits from a central source (store, headman, etc) to vaccinate my own dogs. | ORV_pickup_baits | Radio Button | Yes | Adult family member | adult | NA |
| I would also pick up baits from a central source (store, headman, etc) to vaccinate my own dogs. | ORV_pickup_baits | Radio Button | Yes | Both | both | NA |
| What is best for you to get your dogs vaccinated? | best_method | Radio Button | Yes | At vaccination point during a targeted campaign | campaign | NA |
| What is best for you to get your dogs vaccinated? | best_method | Radio Button | Yes | At a cattle crush pen along with cattle vaccinations | crush_pen | NA |
| What is best for you to get your dogs vaccinated? | best_method | Radio Button | Yes | At veterinary clinic/extension centres | extension_centre | NA |
| What is best for you to get your dogs vaccinated? | best_method | Radio Button | Yes | At a private veterinary clinic | private_vet | NA |
| What is best for you to get your dogs vaccinated? | best_method | Radio Button | Yes | I don't know | no_preference | NA |
| What is best for you to get your dogs vaccinated? | best_method | Radio Button | Yes | At home/village with an oral bait | oral_bait | NA |
| What is best for you to get your dogs vaccinated? | best_method | Radio Button | Yes | at home/village with a parenteral (needle + syringe) vaccine | home_needle | NA |
| Do you know of human rabies cases in your village during 2023 or 2024? | rabies_incidence_humans | Radio Button | Yes | Yes | yes | 2024 \| 2023 |
| Do you know of human rabies cases in your village during 2023 or 2024? | rabies_incidence_humans | Radio Button | Yes | No | no | NA |
| Do you know of human rabies cases in your village during 2023 or 2024? | rabies_incidence_humans | Radio Button | Yes | I don't remember | no_memory | NA |
| Do you know of rabies cases in animals, e.g. dogs in your village during the past year? | rabies_incidence | Radio Button | Yes | Yes | yes | NA |
| Do you know of rabies cases in animals, e.g. dogs in your village during the past year? | rabies_incidence | Radio Button | Yes | No | no | NA |
| Do you know of rabies cases in animals, e.g. dogs in your village during the past year? | rabies_incidence | Radio Button | Yes | I don't remember | no_memory | NA |
| What was the cause for these animal rabies case? | rabies_animals_cause | Radio Button | Yes | Dog bite | dog_bite | 2024 \| 2023 |
| What was the cause for these animal rabies case? | rabies_animals_cause | Radio Button | Yes | Bite from wildlife | wildlife_bite | NA |
| What was the cause for these animal rabies case? | rabies_animals_cause | Radio Button | Yes | I don't remember | no_memory | NA |
| Have you OR any of your household members been bitten by a dog in the last year? | bite_experience | Radio Button | Yes | Yes | yes | Pet/owned dog \| Ownerless/stray dog \| I don't know \| orally vaccinated dog (within 6h after vaccination) |
| Have you OR any of your household members been bitten by a dog in the last year? | bite_experience | Radio Button | Yes | No | no | NA |
| Was it a provoked bite? | bite_provocation | Radio Button | Yes | Yes | yes | NA |
| Was it a provoked bite? | bite_provocation | Radio Button | Yes | No | no | NA |
| Was it a provoked bite? | bite_provocation | Radio Button | Yes | I don't remember | no_memory | NA |
| Was the biting dog vaccinated against rabies? | bite_dog_vaccinated | Radio Button | Yes | Yes (only when verified by certificate) | yes | NA |
| Was the biting dog vaccinated against rabies? | bite_dog_vaccinated | Radio Button | Yes | No | no | NA |
| Was the biting dog vaccinated against rabies? | bite_dog_vaccinated | Radio Button | Yes | I don't know | unknown | NA |
| What happened to the biting dog? | bite_dog_outcome | Radio Button | Yes | Dog quarantined and alive after 10 days | quarantine_survival | NA |
| What happened to the biting dog? | bite_dog_outcome | Radio Button | Yes | Dog quarantined and died within 10 days | quarantine_death | NA |
| What happened to the biting dog? | bite_dog_outcome | Radio Button | Yes | Dog was killed | killed | NA |
| What happened to the biting dog? | bite_dog_outcome | Radio Button | Yes | Dog was not quarantined and died within 10 days after bite | died | NA |
| What happened to the biting dog? | bite_dog_outcome | Radio Button | Yes | Dog was chased away | chased_away | NA |
| What happened to the biting dog? | bite_dog_outcome | Radio Button | Yes | Dog disappeared | disappeared | NA |
| What happened to the biting dog? | bite_dog_outcome | Radio Button | Yes | Dog slaughtered for meat | slaughtered | NA |
| What happened to the biting dog? | bite_dog_outcome | Radio Button | Yes | I don't know | unknown | NA |
| In case the dog died, was a diagnostic test performed? | diagnostics | Radio Button | No | Yes - it was positive | yes_positive | NA |
| In case the dog died, was a diagnostic test performed? | diagnostics | Radio Button | No | Yes - it was negative | yes_negative | NA |
| In case the dog died, was a diagnostic test performed? | diagnostics | Radio Button | No | Not tested | not_tested | NA |
| In case the dog died, was a diagnostic test performed? | diagnostics | Radio Button | No | I don't know | unknown | NA |
| When did you/the bite victim visit a hospital and/or consult a doctor? | hospital_visit | Radio Button | Yes | Never | never | NA |
| When did you/the bite victim visit a hospital and/or consult a doctor? | hospital_visit | Radio Button | Yes | On the same day | same_day | NA |
| When did you/the bite victim visit a hospital and/or consult a doctor? | hospital_visit | Radio Button | Yes | On the next day | next_day | NA |
| When did you/the bite victim visit a hospital and/or consult a doctor? | hospital_visit | Radio Button | Yes | Within a week | within_week | NA |
| When did you/the bite victim visit a hospital and/or consult a doctor? | hospital_visit | Radio Button | Yes | After 1 week | one_week | NA |
| When did you/the bite victim visit a hospital and/or consult a doctor? | hospital_visit | Radio Button | Yes | After 2 weeks | two_weeks | NA |
| When did you/the bite victim visit a hospital and/or consult a doctor? | hospital_visit | Radio Button | Yes | I don't remember | no_memory | NA |
| Did you/the bite victim receive the anti-rabies vaccine? | pep_given | Radio Button | Yes | Yes | yes | NA |
| Did you/the bite victim receive the anti-rabies vaccine? | pep_given | Radio Button | Yes | No | no | NA |
| Did you/the bite victim receive the anti-rabies vaccine? | pep_given | Radio Button | Yes | I don't remember | no_memory | NA |

**Table S2:**

Raw data on oral vaccinations as recorded using the WVS data collection app (<https://missionrabies.com/app/>). The data of this study are openly available in Zenodo at https://zenodo.org/, reference number 10.5281/zenodo.14744857.

**Table S3:**

Raw data on post vaccination monitoring/survey using the WVS data collection app (<https://missionrabies.com/app/>). The data of this study are openly available in Zenodo at https://zenodo.org/, reference number 10.5281/zenodo.14744857.

**Table S4:**

Details on the studies used to compare the vaccination efficiencies (dogs vaccinated per team and day).

| **Study** | **Country** | **Vaccine** | **Details** | **DOI_PMCID** | **Data_1** | **Data_2** | **Data_3** | **Data_4** | **Data_5** | **Data_6** | **Data_7** | **Data_8** | **Data_9** | **Data_10** | **Data_11** |
| --- | --- | --- | --- | --- | --- | --- | --- | --- | --- | --- | --- | --- | --- | --- | --- |
| Zambezi 2024 (ORV, Rabitec) | Namibia | oral | this study | NA | 211,75 | 152,75 | 137,75 | 140,75 | 151,00 | 161,50 | 119,50 | 143,50 | 203,50 | 218,50 | NA |
| Nairobi, Kenya (Perry et al., 1995) | Kenya | parenteral | NA | doi:10.1016/0167-5877(94)00407-A | 28,00 | 43,00 | NA | NA | NA | NA | NA | NA | NA | NA | NA |
| Haiti, (Monroe et al., 2021) | Haiti | parenteral | NA | doi:10.1016/j.actatropica.2016.11.005. | 27,00 | 42,00 | NA | NA | NA | NA | NA | NA | NA | NA | NA |
| Cambodia (Tazawa et al., 2024) | Cambodia | parenteral | NA | doi:10.3390/ani14182654. | 62,00 | NA | NA | NA | NA | NA | NA | NA | NA | NA | NA |
| N'Djamena, Chad (Anyiam et al., 2016) | Chad | parenteral | NA | doi:10.1016/j.actatropica.2016.11.005. | 100,00 | NA | NA | NA | NA | NA | NA | NA | NA | NA | NA |
| Bhutan (Tenzin et al., 2012) | Bhutan | parenteral | NA | doi:10.1016/j.vaccine.2012.05.023. | 100,00 | NA | NA | NA | NA | NA | NA | NA | NA | NA | NA |
| Blantyre, Malawi (Gibson et al., 2016) | Malawi | parenteral | NA | doi:10.1371/journal.pntd.0004824. | 220,00 | NA | NA | NA | NA | NA | NA | NA | NA | NA | NA |
| rural Zambia (Misapa et al., 2024) | Zambia | parenteral | NA | doi:10.3390/tropicalmed9070161. | 8,00 | 10,00 | NA | NA | NA | NA | NA | NA | NA | NA | NA |
| Malawi (Soriano et al., 2020) | Malawi | parenteral | NA | doi:10.1371/journal.pntd.0008004 | 37,20 | 58,80 | 27,10 | 75,90 | 39,90 | 73,10 | 41,50 | 53,90 | 19,20 | 77,90 | 33,00 |
| rural Kenya (Ferguson et al., 2020) | Kenya | parenteral | NA | doi:10.1371/journal.pntd.0008260. | 54,80 | NA | NA | NA | NA | NA | NA | NA | NA | NA | NA |
| Lima, Peru (Chomel et al., 1988) | Peru | parenteral | NA | doi:10.1093/clinids/10.Supplement\_4.S697 | 80,05 | NA | NA | NA | NA | NA | NA | NA | NA | NA | NA |
| rural Namibia (Athingo et al., 2020a) | Namibia | parenteral | NA | doi:10.3390/tropicalmed5010012. | 78,91 | 76,98 | 77,69 | 79,29 | 53,91 | 71,76 | 84,58 | 6,68 | NA | NA | NA |
| rural Namibia (Athingo et al., 2020b) | Namibia | parenteral | NA | doi:10.1371/journal.pntd.0008948. | 37,00 | NA | NA | NA | NA | NA | NA | NA | NA | NA | NA |
| rural Namibia (NCA 2024) | Namibia | parenteral | vaccination campaign 2024 | NA | 51,00 | 78,00 | 37,00 | 66,00 | 20,00 | 36,00 | 84,00 | NA | NA | NA | NA |
| Mali (Muthiani et al., 2015) | Mali | parenteral | NA | doi:10.1016/j.prevetmed.2015.04.007. | 12,90 | NA | NA | NA | NA | NA | NA | NA | NA | NA | NA |
| N'Djamena, Chad (Dürr et al., 2009) | Chad | parenteral | NA | doi:10.1017/S0950268809002386. | 11,70 | NA | NA | NA | NA | NA | NA | NA | NA | NA | NA |
| N'Djamena, Chad 2012 (Lechenne et al. 2016 ) | Chad | parenteral | NA | doi:10.1016/j.vaccine.2015.11.033. | 53,00 | 70,00 | NA | NA | NA | NA | NA | NA | NA | NA | NA |
| N'Djamena, Chad (Kayali et al., 2003) | Chad | parenteral | NA | PMCID: PMC2572337 | 50,80 | NA | NA | NA | NA | NA | NA | NA | NA | NA | NA |
| Zambia (Chazya et al., 2024) | Zambia | parenteral | NA | doi:10.3389/fvets.2024.1492418 | 71,00 | 143,00 | NA | NA | NA | NA | NA | NA | NA | NA | NA |
